# Prenatal Alcohol Exposure and Child Psychosocial Behavior: A Sibling Fixed-Effects Analysis

**DOI:** 10.3389/fpsyt.2018.00570

**Published:** 2018-11-06

**Authors:** Kayoko Ichikawa, Takeo Fujiwara, Ichiro Kawachi

**Affiliations:** ^1^Department of Health Informatics, Kyoto University School of Public Health, Kyoto, Japan; ^2^Department of Global Health Promotion, Tokyo Medical and Dental University, Tokyo, Japan; ^3^Department of Social and Behavioral Sciences, Harvard T.H. Chan School of Public Health, Boston, MA, United States

**Keywords:** alcohol-related disorders, developmental disabilities, maternal-fetal relations, pregnancy, prenatal alcohol exposure

## Abstract

**Background:** The association between low levels of alcohol consumption during pregnancy and children's health remains unclear because of the difficulty in ruling out residual genetic and environmental confounding factors. In this study, using a within-family sibling fixed effects design, we sought to examine the association between low prenatal alcohol exposures (PAE) and children's overall psychosocial behavior in a Japanese cohort.

**Methods:** We used maternal and sibling data from the Japanese Study of Stratification, Health, Income and Neighborhood 2012-2013. Households were recruited from the Tokyo metropolitan area through clustered random sampling. Children under 18 years old who have siblings (*n* = 1,600) and their mothers were selected. PAE status was retrospectively measured, and classified by binominal and continuous measurements. Outcome measures of children's psychosocial behavior were assessed with the Child Behavior Checklist T-score.

**Results:** Low PAE was significantly associated with the offspring's anxiety problems (β = 1.54, 95%CI = 0.26, 2.82) and internalizing problems (β = 2.73, 95%CI = 0.87, 4.60), and marginally significant with the offspring's total problem scores (β = 2.34, 95%CI = −0.24, 4.92). There was no significant difference in PAE between boys and girls when it comes to behavioral problems.

**Conclusions:** Low PAE was associated with children's anxiety, internalizing problems and overall problems, taking into account possible unobserved genetic and environmental confounding influences.

## Introduction

The U.S. Centers for Disease Control, the U.S. Surgeon General, the American College of Obstetricians and Gynecologists, and the American Academy of Pediatrics uniformly advise women not to consume alcohol during pregnancy (1–5). However, the debate on whether “moderate consumption” of alcohol can be safely practiced during pregnancy continues (6–9). For example, a large-scale study in Denmark recently reported that antenatal exposure to binge drinking was associated with internalizing and externalizing behavioral problems among boys (5). However, the study found no association with lower doses of alcohol exposure. In fact, children of expectant mothers who consumed moderate amounts of alcohol were found to have better mental health than those whose mother abstained from alcohol–a controversial result that was widely reported in the media.

Although it is widely accepted that binge drinking during pregnancy increases the risk of fetal alcohol syndrome (10, 11), whether low-to-moderate levels of alcohol consumption during pregnancy can affect children's health remains controversial (12–14). A recent meta-analysis which assessed both normal and abnormal development as continuous variables concluded that mild-to-moderate prenatal alcohol exposure (PAE) during all trimesters was not associated with child psychosocial outcomes such as cognition and mental development (12). However, it suggested that further studies were needed to rule out residual confounding factors.

There are several reasons why it is challenging to investigate the effects of PAE on children's behavioral problems. First, mothers tend to under report their drinking levels during pregnancy because of prevailing social norms (14). Second, any correlation between maternal drinking during pregnancy and child outcomes is likely to be confounded by a range of unobserved factors including family environment and shared genetic influences. Third, it is ethically infeasible to design a randomized controlled trial to investigate the association between PAE and child behavioral outcomes. As an alternative to directly manipulating the exposure of interest, some sort of quasi-experimental approach is needed, such as the within-family sibling fixed-effects design. To our knowledge, there is a limited number of studies that have used quasi-experimental design to infer causality of deferential low PAE on behavior problems among twins, siblings, or cousins (15, 16). Hence, there has been a call for the implementation of more quasi-experimental study using natural experiment (17).

Notably, studies that considered observed and unobserved confounding factors are rare (16, 18, 19). The basic idea of a sibling fixed-effects design is to leverage the within-family, between-sibling differences in exposure to maternal drinking during pregnancy (e.g., mother drank during first pregnancy, but not during subsequent pregnancies), which effectively differentiates sibling-invariants observed and unobserved confounding variables, such as genetic influences or maternal temperament/personality, or family environment (19, 20).

The findings from the few existing studies using the sibling fixed-effects approach (16, 18, 19) suggest that even low-to-moderate PAE is a risk factor for children's externalizing problems such as attention deficit hyperactivity disorder, infant difficulties and children's behavioral problems. However, the literature remains sparse, especially in the Asian context where alcohol consumption among women is low compared to Western societies (21).

In this study, we examined the association between low PAE and children's overall psychosocial behavior in a Japanese cohort using the sibling fixed-effects design.

## Methods

### Participants

The J-SHINE (Japanese study of Stratification, Health, Income and Neighborhood) is an ongoing cohort study established since 2010. Details of the study have been previously described (22). Briefly, the baseline survey was carried out between 2010 and 2011, when a clustered random sample of individuals aged 25–50 years residing in four municipalities in urban or suburban settings of the Tokyo metropolitan area were invited to participate. The household survey asked about the health of all children under the age of 18 years co-residing with the subjects. A follow-up survey was conducted between 2012 and 2013. In the baseline survey, 13,920 individuals were randomly selected from the “*koseki*” registration system, a compulsory domiciliary registration system in Japan that included all residents in target area (*N* = 594,249). Among the individuals invited to participate, 4,385 men and women responded (31.6% response rate), including 2,184 households with children under 18 years. By wave 2, the number of households with children increased to 2,244, and of these, 1,520 households (67.7%) agreed to participate in the follow-up survey (including 2,470 children under 18 years). Written informed consent was received from all the participants in the study. We excluded children who did not have the outcome data and the prenatal mothers who drink alcohol more than 2 times per week (*N* = 33, 1.7% of all sample) because we focused on low-level drinkers. Therefore, the number of children in the study was 1,933. In addition, we used only sibling data for the fixed-effects models, the total number of children who had siblings and who had outcome data was 1,600 (mean age 114.1 months ± 52.5 *SD*). Among them, 1,046 had two siblings, 518 had three siblings, and 36 had four siblings. If the number of siblings was more than two in one family, we compared each sibling pair separately. For example, if the number of the children were three (A, B, C) in one household, we compared A-B, A-C, and B-C.

The J-SHINE was conducted using computer-assisted personal interviewing (CAPI), unless the participants requested a face-to-face interview. This study was carried out in accordance with the recommendations of ethical guidelines for medical and health research involving human subjects. The study protocol was approved by the ethics committee of the Graduate School of Medicine of the University of Tokyo. All subjects provided written informed consent in accordance with the Declaration of Helsinki.

## Measurements

### Prenatal alcohol drinking

The J-SHINE study asked mothers to report their drinking behavior during each pregnancy retrospectively. Response categories included: (1) 2 times or more per week, (2) 1–4 times per month, (3) rarely (but not zero), and (4) never. We excluded those who responded with “2 times or more per week” to focus on low prenatal alcohol drinking during pregnancy, and combined “1–4 times per month” and “rarely,” so that PAE was binarized into “never” vs. “ever.”

### Outcome variable: the CBCl4-18/2-3 (child behavior check list 4-18/2-3)

The outcome variable, that is, children's psychosocial developmental problems, was assessed with the CBCL4-18 checklist which targets children aged 4 to 18 years, and the CBCL2-3 which targets children aged 2 to 3 years (23, 24). The CBCL is a scale that assesses internalizing, externalizing, and total behavior problems using 113 items, with good psychometric properties (24). A higher score denotes more problematic behavior. Ratings were completed by caregivers (mother, *n* = 1316, 83.7%). The T score of each CBCL score was calculated using the standardized distribution among Japanese children and mean score represents the 50th percentile, which has been shown to have good reliability and validity (23–25).

### Covariates

We considered a wide range of potential correlates of PAE as control variables in adjusted models. There are two types of covariates: sibling variables that are less likely to be variant and sibling-varying variables. With respect to our sibling fixed-effects models, sibling variables that are less likely to be variant include factors that did not vary between siblings, that is, parent's age, educational level, working status, family income and domestic violence and others that are less likely to be changeable variables. In contrast, sibling-varying variables include children's age, sex, and prenatal smoking during each pregnancy.

### Statistical analysis

To estimate the relationship between maternal drinking during pregnancy and children's behavioral outcomes, we conducted ordinary least squares (OLS) regressions controlling for all observed sibling-varying and sibling-invariant variables for the full sample, as well as for the sibling sub-sample. Next, we conducted sibling fixed-effects analysis only among the siblings (i.e., singletons do not contribute to this analysis), with further adjustment of correlation of 3 or more siblings comparisons from the same family. In the fixed-effects model, the effects of PAE on children's outcomes were calculated by differentiating the unobserved, sibling-invariant shared factors, such as genetic or family environmental influences. Finally, we conducted fixed-effects models stratified by child sex (i.e., male-male, female-female, and male-female pair of siblings) as a sensitivity analysis. All analyses were performed with STATA 13.0 (StataCorp LP, College Station, TX, USA).

## Results

Table 1 shows the demographic characteristics of families and children comparing the full sample (*n* = 1,933) and the sample restricted to the siblings (*n* = 1,600). In the full sample, the frequencies of prenatal alcohol “1-4 times/month,” “rarely,” and “never” were 6.1, 21.7, and 55.4%, respectively. The vast majority of pregnant women in this Japanese cohort therefore fitted into the abstention or light drinking categories.

**Table 1:** Demographic characteristics of children and families for individual level.

**Variables**	**All sample (*n* = 1933)**	**Siblings sample (*n* = 1600)**
	***n* (%)**	***n* (%)**
**AREA CHARACTERISTICS**		
Tokyo urban area (Adachi)	393 (20.3)	332 (20.8)
Tokyo urban area (Mitaka)	389 (20.1)	306 (19.1)
Tokyo suburban area (Kashiwa)	646 (33.4)	546 (34.1)
Tokyo suburban area (Tokorozawa)	505 (26.1)	416 (26.0)
**FAMILY CHARACTERISTICS**		
Mother's age (years old) (mean, SD)	37.5 (5.5)	37.2 (5.3)
Father's age (years old) (mean, SD)	39.4 (6.3)	39.2 (6.2)
**MOTHER'S EDUCATION**		
< = 12 years	946 (49.0)	388 (24.3)
>12 years	875 (45.3)	1186 (74.1)
unknown	112 (5.8)	26 (1.6)
**FATHER'S EDUCATION**		
< = 12 years	427 (22.1)	371 (23.2)
>12 years	1441 (74.6)	1181 (77.8)
unknown	30 (1.6)	48 (3.0)
Working mother	875 (45.3)	707 (44.2)
Working father	1631 (84.4)	1363 (85.2)
**NUMBERS OF FAMILY**		
< = 4	1351 (60.0)	1054 (65.9)
> = 5	574 (27.0)	541 (33.8)
Unknown	8 (0.4)	5 (0.3)
**FAMILY INCOME**		
< JPY 5 million^a^	438 (22.7)	362 (22.6)
JPY 5–7.5 million	572 (30.0)	484 (52.9)
JPY 7.5–10 million	391 (20.2)	329 (20.6)
>JPY 10 million	369 (19.1)	288 (18.0)
unknown	163 (8.4)	137 (8.6)
Domestic violence	631 (32.6)	538 (33.6)
Prenatal smoking	67 (3.5)	59 (3.7)
**CHILDREN'S CHARACTERISTICS**		
**Number of siblings**		
1	333 (17.3)	**–**
2	1046 (54.1)	1046 (65.4)
3	518 (26.8)	518 (32.4)
4	36 (1.9)	36 (2.3)
Sex (male)	971 (50.2)	800 (50.0)
Age (months) (mean, SD)	113.9 (53.7)	114.1 (52.5)
**PRENATAL ALCOHOL CONSUMPTION**		
1-4 times/month	110 (5.7)	98 (6.1)
rare	401 (20.7)	347 (21.7)
never	1083 (56.0)	886 (55.4)
unknown	339 (17.5)	269 (16.8)
**CBCL** ***T*** **SCORE**, ***N*****, MEAN (*****SD*****)**		
**4-18 Years old**		
Physical problem	52.3 (4.8)	52.1 (4.7)
Social problem	53.3 (5.3)	53.0 (5.1)
Thought problem	51.0 (3.8)	51.0 (3.8)
Delinquency	52.9 (5.2)	52.9 (5.2)
**2-18 YEARS OLD**		
Withdrawal	53.4 (5.4)	53.2 (5.4)
Anxiety problem	52.7 (4.9)	52.6 (4.9)
Attention problem	53.0 (5.7)	52.8 (5.6)
Aggressiveness	53.4 (5.5)	53.4 (5.5)
Internalizing problem	49.6 (7.8)	49.3 (7.8)
Externalizing problem	49.6 (8.4)	49.5 (8.4)
Total problem	48.4 (9.7)	48.0 (9.7)

a *JPY, Japanese Yen; JPY 120 is approximately equal to US 1 dollar*.

There was a total of 491 sibling pairs whose mother never drank during pregnancies (Appendix 1). Further, 37 (4.6%) mothers reported drinking during their first pregnancy but not during subsequent pregnancies. Another 31 mothers (3.9%) reported abstaining from alcohol during their first pregnancy, but consumed alcohol during subsequent pregnancies. Effects of PAE, binarized according to “ever” vs. “never,” are shown in Table 2. In the OLS model, the effect of PAE was not significantly associated with children's psychosocial problems in both the overall sample and the sibling sample. In the unadjusted sibling fixed-effects model, prenatal drinking was significantly associated with children's delinquency (β = 1.63, 95%CI = 0.02, 3.25), anxiety problems (β = 1.50, 95%CI = 0.15, 2.85), internalizing problems (β = 2.62, 95%CI = 0.61, 4.62), and total problem scores (β = 2.39, 95%CI = 0.11, 4.66). After adjusting for sibling-varying variables, maternal drinking during pregnancy was significantly associated with offspring's anxiety problems (β = 1.54, 95%CI = 0.26, 2.82), internalizing problems (β = 2.73, 95%CI = 0.87, 4.60) and marginally significant with offspring's overall problem scores (β = 2.34, 95%CI = −0.24, 4.92). Additionally, adjusting for sibling-varying and sibling-invariant variables, such as parent's working status and maternal drinking during pregnancy, remained significantly associated with children's anxiety problems, internalizing problems and overall problem scores. In sensitivity analysis, maternal drinking during pregnancy measured with continuously (i.e., frequencies of alcohol drinking per month) was not statistically significant (Appendix 2).

**Table 2:** OLS models and fixed-effects models of the association between PAE^a^ and children's psychosocial behaviors.

	**All sample, OLS, all measured variables adjusted^b^**		**Siblings sample, OLS, all measured variables adjusted^b^**		**Fixed effects model,unadjusted**		**Fixed effects model, adjusted^c^**		**Fixed effects model, adjusted^d^**	
**CBCL T score**	**β**	**95%CI**	**β**	**95%CI**	**β**	**95%CI**	**β**	**95%CI**	**β**	**95%CI**
**4-18 YEARS OLD**										
Physical problem	0.29	−0.28, 0.86	0.51	−0.10, 1.11	0.94	−0.68, 2.56	1.19	−1.74, 4.11	0.90	−1.85, 3.65
Social problem	0.12	−0.53, 0.77	0.47	−0.20, 1.14	0.74	−0.95, 2.44	1.30	−0.63, 3.23	1.22	−0.76, 3.20
Thought problem	−0.35	−0.77, 0.07	−0.24	−0.67, 0.20	−0.83	−2.11, 0.44	−0.80	−3.34, 1.73	−0.86	−3.35, 1.63
Delinquency	0.48	−0.14, 1.10	0.51	−0.17, 1.19	**1.63**	**0.02, 3.25**	1.73	−0.33, 3.79	1.57	−0.50, 3.64
**2–18 YEARS OLD**										
Withdrawal	−0.01	−0.65, 0.62	0.19	−0.50, 0.88	−0.51	−1.95, 0.93	−0.40	−1.59, 0.79	−0.41	−1.66, 0.84
Anxiety problem	0.14	−0.43, 0.70	0.43	−0.18, 1.05	**1.50**	**0.15, 2.85**	**1.54**	**0.26, 2.82**	**1.40**	**0.09, 2.72**
Attention problem	0.27	−0.37, 0.91	0.43	−0.23, 1.10	−0.53	−2.20, 1.13	−0.44	−2.57, 1.69	−0.58	−2.78, 1.63
Aggressiveness	0.32	−0.33, 0.96	0.49	−0.21, 1.20	1.18	−0.45, 2.80	1.15	−0.70, 2.99	1.23	−0.69, 3.16
Internalizing problem	0.41	−0.49, 1.31	0.91	−0.07, 1.88	**2.62**	**0.61, 4.62**	**2.73**	**0.87, 4.60**	**2.68**	**0.73, 4.63**
Externalizing problem	0.49	−0.48, 1.45	0.72	−0.35, 1.78	1.77	−0.45, 3.99	1.50	−1.04, 4.04	1.64	−0.94, 4.22
Total problem	0.42	−0.71, 1.55	0.95	−0.30, 2.19	**2.39**	**0.11, 4.66**	2.34^e^	−0.24, 4.92	2.32	−0.34, 4.98

a *Measurements of alcohol drinking is binary outcomes (never = 0, more than one time = 1)*.

b *Adjusted by children's age and sex, parent's age, education and working status, family income, prenatal smoking, domestic violence, clustered by family ID*.

c *Adjusted by children's age and sex and difference levels of prenatal smoking baseline prenatal drinking status among siblings, clustered by family ID*.

d *Adjusted by children's age and sex, parent's education level, family income, family number, domestic violence and baseline prenatal drinking status among siblings, clustered by family ID*.

e *Marginally significant: p = 0.08*.

*Bold values means the results which is statistically significant*.

Table 3 shows the sibling fixed-effects results of maternal drinking and children's psychosocial problems, stratified by children's sex. Drinking during pregnancy was not associated with girl's thought problems (β = −2.56, 95%CI = −7.59, 2.47) attention problems (β = −0.16, 95%CI = −2.29, 1.96), anxiety problems (β = −2.56, 95%CI = −0.80, 3.07) and aggression (β = 0.27, 95%CI = −3.17, 3.70). When it comes to the effect of drinking during pregnancy on internalizing, externalizing and total problems, there was no different between boys and girls.

**Table 3:** Fixed-effects model stratified by sibling's sex pairs.

	**Male-male pair**		**Female-female pair**		**Male-female pair**	
**CBCL T score**	**β**	**95%CI**	**β**	**95%CI**	**β**	**95%CI**
**4-18 YEARS OLD**						
Physical problem	0.01	−3.88, 3.90	1.05	−4.41, 6.51	2.11	−1.95, 6.18
Social problem	1.34	−2.28, 4.97	1.16	−1.64, 3.96	1.27	−1.71, 4.24
Thought problem	1.39	−1.35, 4.12	−2.56	−7.59, 2.47	−1.43	−4.13, 1.27
Delinquency	0.76	−3.22, 4.75	0.16	−3.06, 3.39	**3.15**	**0.52, 5.77**
**2–18 YEARS OLD**						
Withdrawal	−0.81	−3.41, 1.79	−1.22	−2.47, 0.03	0.33	−1.28, 1.95
Anxiety problem	2.32	−0.56, 5.20	1.13	−0.80, 3.07	1.37	−0.07, 2.82
Attention problem	1.04	−1.98, 4.06	−0.16	−2.29, 1.96	−1.60	−5.49, 2.30
Aggressiveness	1.27	−2.77, 5.30	0.27	−3.17, 3.70	1.68	−0.86, 4.22
Internalizing problem	2.35	−1.76, 6.46	2.32	−1.18, 5.81	**3.34**	**1.05, 5.63**
Externalizing problem	0.86	−3.96, 5.67	−0.10	−4.37, 4.18	2.76	−1.18, 6.69
Total problem	1.24	−3.24, 5.72	0.64	−3.26, 4.54	4.03	−0.05, 8.11

*Bold values means the results which is statistically significant*.

## Discussion

We found that low PAE during pregnancy is associated with children's anxiety, internalizing and overall problems. Our findings serve as additional evidence for the deleterious impact of maternal drinking during pregnancy on children's psychosocial behavior, and are consistent with previous animal models suggesting that even a relatively small amount of alcohol during fetal development can result in an increase in synaptic connectivity specific to the basolateral amygdala and induce a subtle anxiety-like behavior in rats (26).

Previous studies have reported inconsistent results regarding which sex is more affected by exposure to alcohol *in utero* (5, 27). Sayal et al. suggested that girls whose mother drank alcohol less than once per week during pregnancy have increased risk of behavioral problems; however, these results should be interpreted with caution because of a lack of evidence on dose-response effects (27). In contrast, another study indicated that boys are more vulnerable to PAE than girls (5), because of the brain development trajectory (28, 29). Our findings suggest that PAE is particularly deleterious for certain developmental problems such as attention problems, anxiety problems and aggression in male offspring. However, when it comes to internalizing, externalizing and overall developmental problems, there is no difference between sexes. The mechanism of how PAE affects children's behavior is still unknown and we cannot eliminate social environment completely in this study.

Our study has several limitations. First, the assessment of drinking during pregnancy was retrospectively self-reported by the mothers. Furthermore, we assessed only the frequency of drinking (as opposed to the total amount of ethanol consumed) or the timing of drinking during different trimesters of pregnancy. However, there is evidence that retrospective reports of prenatal substance use can be reliable (30, 31). Nonetheless, further study on the effects of both the timing of alcohol use and the total amount consumed is warranted. Second, the assessment of child psychosocial problems was reported by the parents, which may have given rise to information bias. However, in the sibling fixed-effects model, reports on behavior problems among siblings were made by the same parent, thereby reducing the possibility of differential misclassification. It has also been found by independent researchers that assessment by mothers is often more reliable than laboratory assessment because the latter is only a snapshot of children's behaviors (32). Finally, the sibling fixed-effects models cannot completely control for unknown sibling-varying confounders such as changes in family circumstances and social situations that differed between siblings. Although this study used sibling-invariant variables as parent's age, educational level, working status, family income and domestic violence, these factors could vary between siblings and we did not measure these variables at the time when the mothers became pregnant. Nonetheless, fixed-effects models are known to provide a more credible causal identification strategy compared with traditional OLS regression models (20, 33).

In conclusion, our study provides additional evidence that even low PAE during pregnancy may adversely affect children's psychosocial behaviors, especially anxiety problems. Our findings provide further support for the current recommendation of abstinence during pregnancy, based on the notion that there is no known safe threshold of alcohol consumption during pregnancy.

## Author contributions

KI analyzed the data and drafted the paper and designed the study. IK was responsible for conception and design of the study and coauthored the paper, TF was involved in the study design, supervised the data analysis, and coauthored the paper. All authors read and approved the final manuscript.

### Conflict of interest statement

The authors declare that the research was conducted in the absence of any commercial or financial relationships that could be construed as a potential conflict of interest.

## Abbreviations

PAE: prenatal alcohol exposure
OLS: ordinary least squares.

## Appendix

**Table A1:** Distribution of prenatal alcohol consumption among siblings pair (pair = 799) N (%).

**Both siblings were not exposed to alcohol**	**491 (61.5)**
**Senior child exposed, junior child not exposed**	**37 (4.6)**
**Senior child not exposed, junior child exposed**	**31 (3.9)**
**Both siblings were exposed to alcohol**	**240 (30.0)**

**Table A2:** OLS models and fixed-effects models of the association between PAE^a^ and children's psychosocial behaviors.

	**All sample, OLS, all measured variables adjusted****^b^**		**Siblings sample, OLS, all measured variables adjusted****^b^**		**Fixed effects model,unadjusted**		**Fixed effects model, adjusted****^c^**		**Fixed effects model, adjusted****^d^**	
**CBCL T score**	**β**	**95%CI**	**β**	**95%CI**	**β**	**95%CI**	**β**	**95%CI**	**β**	**95%CI**
**4-18 YEARS OLD**										
Physical problem	0.25	–0.26, 0.76	0.53	–0.02, 1.07	1.25	–0.09, 2.60	1.47	–0.34, 3.27	1.10	–0.47, 2.67
Social problem	0.34	–0.24, 0.92	0.54	–0.06, 1.13	0.76	–0.64, 2.16	0.94	–1.01, 2.89	0.82	–1.22, 2.86
Thought problem	–0.20	–0.58, 0.18	–0.11	–0.50, 0.28	–0.49	–1.55, 0.56	–0.37	–2.20, 1.47	–0.52	–2.41, 1.36
Delinquency	0.47	–0.09, 1.03	0.47	–0.14, 1.08	1.16	–0.18, 2.50	1.12	–0.16, 2.41	0.85	–0.49, 2.20
**2–18 YEARS OLD**										
Withdrawal	0.17	–0.39, 0.73	0.45	–0.16, 1.07	–0.72	–1.92, 0.49	–0.50	–1.62, 0.61	–0.62	–1.78, 0.53
Anxiety problem	0.17	–0.33, 0.67	0.47	–0.08, 1.01	0.49	–0.65, 1.62	0.57	–0.87, 2.01	0.41	–1.00, 1.82
Attention problem	0.50	–0.07, 1.06	**0.68**	**0.09, 1.27**	0.37	–1.03, 1.77	0.51	–1.36, 2.37	0.29	–1.70, 2.29
Aggressiveness	**0.69**	**0.12, 1.25**	**0.93**	**0.31, 1.54**	0.74	–0.61, 2.08	0.74	–1.02, 2.51	0.64	–1.16, 2.44
Internalizing problem	0.58	–0.22, 1.38	**1.08**	**0.21, 1.95**	1.02	–0.67, 2.71	1.35	–0.18, 2.87	1.25	–0.30, 2.80
Externalizing problem	**1.14**	**0.28, 1.99**	**1.35**	**0.41, 2.29**	1.22	–0.65, 3.08	1.39	–0.83, 3.62	1.36	–0.89, 3.60
Total problem	**1.01**	**0.01, 2.01**	**1.42**	**0.31, 2.52**	1.12	–0.79, 3.04	1.31	–0.76, 3.38	1.14	–1.01, 3.30

a *Measurements of alcohol drinking was frequencies of alcohol drinking per month (continuous variables)*.

b *Adjusted by children's age and sex, parent's age, education and working status, family income, prenatal smoking, domestic violence, clustered by family ID*.

c *Adjusted by children's age and sex and difference levels of prenatal smoking baseline prenatal drinking status among siblings, clustered by family ID*.

d *Adjusted by children's age and sex, all measured variables about family and baseline prenatal drinking status among siblings, clustered by family ID*.

*Bold values means the results which is statistically significant*.
